# Mr. Earnest, on Burns and Scalds

**Published:** 1807-11

**Authors:** Robert Earnest

**Affiliations:** House Surgeon to the Sheffield General Infirmary


### *To the Editors of the Medical and Physical Journal.*

GENTLEMEN,

IN looking over a few of the last Numbers of your Journal, I met with several papers on the subject of Burns and Scalds, in which there seems a difference of opinion respecting the stimulating and the sedative or cooling mode of treatment; but each remedy is recommended and vindicated by high authority. I beg, through the medium of your very useful publication, to offer a few remarks on the subject, accompanied by a case.

I have been led to the following remarks by the case of a young woman, who was severely scalded with water at the boiling temperature, which was accidently thrown upon her, and a great extent of surface was injured. In this case the early application of the oleum terebinthinæ was essentially useful, as will appear in the history of the case.

Notwithstanding all that has been said in favour of the different remedies used in the cure of burns and scalds, the success attending the treatment of such accidents must depend upon close and minute observation in practice, and the faithful publication of individual cases, which will eventually establish the adoption of either the stimulating, the sedative, or the emollient mode of treatment. It is a pity that senior practitioners should be at the trouble of writing and publishing their disputations, merely for the sake of argument, and of supporting their own individual theories and practices. Would it not be better for those who suffer under the accidents in question, and more honourable to the profession, were they to lay down certain laws or rules for the guidance of the junior and less experienced practitioners?

Before any, either internal or external, remedies are administered, I conceive the following particulars ought to be carefully inquired into. 1. The age and strength of the patient. 2. Peculiarity of constitution. 3. Whether subject to any other malady. 4. Whether of a costive or relaxed habit. 5. If a female, whether it be the menstruating period; or whether she be in a state of pregnancy, and how far advanced. 6. More particularly to ascertain, as near as possible, the nature and degree of the heat, the duration of its application, and also the nature of the parts injured, and the symptoms brought on by their derangement.

I am

I am of opinion that a surgeon cannot, with propriety or professional decorum, apply to a burn or a scald any external remedy, however high its reputation as a specific, without previously informing himself of the above queries; which shews, therefore, the necessity also of attending to the different divisions and degrees of burns, mentioned by Motherby, Heister, Kentish, and others; and of making use of such external remedies as are suitable to the degree of injury the parts may have sustained, and the symptoms produced.

I have frequently remarked, that ulcers which have been the result of a common phlegmon, arising from an internal cause, have required very different treatment in different persons, when, at the same time, the circumstances and symptoms have been apparently analagous to each other. This I apprehend is to be accounted for by the individual peculiarity of constitution, and probable hereditary ailments. And with respect to external applications in cases of burns and scalds, I have known that what has been useful in one case, has been hurtful in another; and so it is in regard to internal remedies.

Practical observation has long ago proved, that different persons, labouring under similar complaints, are differently affected and acted upon by the same class of medicines. Whether this difference of effect be owing to an increased or diminished irritability of the stomach and bowels, or to some peculiar idiosyncrasy, I will not presume to say; I shall therefore leave it for others, more learned on the subject, to investigate and explain.

The following observations will in a great measure prove, what I have before remarked, respecting the effects resulting from any particular class of medicines, when exhibited to different patients, apparently labouring under similar maladies.

One patient in phthisis can bear a large dose of digitalis twice in a day, and is able to persevere in it with advantage, for a considerable length of time; whilst another, apparently in a similar situation, in every respect, can scarcely bear the medicine at all, although given but in very small doses.

One patient in dropsy is able to take, for a considerable length of time, and with the greatest advantage, a bolus composed of calomel and squills, every other night, and in the morning following an ounce of cream of tartar in a state of solution; whilst another, apparently in a similar situation, cannot retain the medicines in the stomach, though the doses shall be considerably smaller. In

In cases of syphilis, I have known some persons salivated with four grains of the calcined mercury; whilst others have taken twenty or upwards before such an effect could be accomplished.

Some diabetic patients can take from 1 to 3 drachms of the diluted nitric acid, in the course of twenty-four hours; whilst others can scarcely bear the effects of one drachm in the same space of time. An instance of this kind occurred not long ago. About a month since I received a letter from a medical friend who resides at Leeds. He was then attending a diabetic patient, and he wrote me the following account of the case, having in recollection at the same time a case of diabetes mellitus, which I published in the Med. and Phys. Journal, vol. 13, page 152, and which was successfully treated by the nitric acid. The account he sent me is as follows. "I have a Case of Diabetes under treatment, in a middle aged, and otherwise healthy male subject, which I was called to about a month ago, when he voided sixteen pints in twenty-four hours. I immediately gave him 1 drachm of the diluted nitric acid each day, which disordered his whole frame exceedingly; increased his thirst, (before very considerable) produced sickness and vomiting, griping and purging. These were, however, relieved by an opiate, and his urine was immediately reduced in quantity one half. I again repeated the acid, but have not been able to get him to take more than 1 drachm and a half of the acid in two days, and from this quantity he complains much at times of the effect. His urine still continues at eight pints, yielding at first real saccharine matter upon inspisation in the proportion of about an ounce to a gill, of the consistence of treacle. At present, perhaps, the complaint may be said to be stationary, and I fear we shall not succeed in a cure so happily as you did."

During my residence at this Infirmary, I have many times had the opportunity of watching the progress and effects resulting from phlegmonous inflammations, which have been brought on in different persons, either by blows, or contusions from other causes. And according to the health and strength of the person, and difference of constitution, different effects have been produced.

The effects of a contusion or blow upon a soft part, are, tumour, hardness, redness, and lividness; which are sooner dispersed, and more resisted in a strong, robust, and healthy man, than in a weakly, delicate, and irritable man. In the former, when inflammation has been in-

 duced duced by such a cause, the vigorous action of the system, along with resolvent local applications, will soon subdue the inflammation and restore the parts to a healthy action: whilst in the latter, the whole frame appears to sympathize with the parts locally affected; and symptomatic fever almost immediately succeeds in such habits, and the parts injured not unfrequently terminate in abscess.

In regard to burns and scalds, if the same degree of heat, either in a solid or a fluid state, be applied to persons with different constitutions, in respect to health and strength, the effects, I am confident, will be more violent in the person with a weakly constitution, than in the other with a strong and vigorous system. And as Dr. Kentish says, "to what are we to attribute the different appearances in these two cases, both arising from the same cause? Is it not natural to conclude, that the different degrees of strength which they possessed was the cause? What conclusion may be drawn from this? Surely, that strength resists the sympathetic irritative actions of other parts, and that weakness favours them."

Of late much has been written by Dr. Kentish and others, in favour of the oleum terebinthinæ, as a specific remedy for burns; and as I have known it to be of the greatest utility in several very severe cases, I must confess myself to be one of the advocates in favour of this remedy, where it is judiciously made use of; but I have seen the turpentine applied in some cases at improper periods, and to very irritable habits, and where it has proved extremely injurious by bringing on much pain, and an increase of inflammation, and which has been the means of retarding the cure, that might have been effected by remedies much more mild.

If the remedial powers of oil of turpentine be so great, is it not probable that the following articles, in proportion to their degrees of volatility, may also be useful in cases of burns and scalds. Æther, spirits of wine and camphor, spirits of wine alone, brandy, rum, and, perhaps, many of the essential oils?

"Dr. Kentish, who has had very extensive practice among the workmen in the coal mines at Newcastle, where explosions often occur, is of opinion that pure alchohol, or oil of turpentine, is the best local application at the beginning, and a cordial diet to support the patient's strength."

Dr. K. must have had frequent opportunities of trying the effects of turpentine in various degrees of injury arising ing from burns, which has proved to him the value of such an external application: and from what he has written and published on the subject, the oleum terebinthinæ is now almost generally adopted as the grand specific remedy for injuries of this kind; and certainly much praise is due to him for urging the adoption of what is called the stimulating plan; but more especially in cases where the injury is very extensive.*

I have frequently remarked that the turpentine as a local dressing, if applied immediately after the accident, is by far the most speedy in mitigating the distressing pain, rigors, and anxiety, with which such patients are afflicted.

When it is thought expedient to make use of the oil of turpentine in cases of burns, my experience tells me that it ought to be applied to the parts injured as early as possible after the accident has happened, and if the consequent inflammation be not removed by resolution in the first instance, the turpentine in a milder form should be continued until signs of suppuration commence, and then it should be changed for a dressing much more mild.

Fearing that I have already trespassed beyond the limits of your publication, I haste now to detail the case I first alluded to.

CASE.——M. B. a girl 18 years of age, low in stature, but is otherwise compact, muscular, and strong. She has been for a considerable length of time troubled with epileptic fits, commonly once or twice in a week, owing probably to her never yet having menstruated. She lives servant with her aunt, who keeps a public house.

In the morning of the 31st of October, 1806, she was assisting in brewing. After she had filled a pail with boiling water, which she rested upon the side of the boiler, and in attempting to lift it upon her head, her foot slipt and she fell down, and the boiling water was turned upon her. The whole of the cuticle, from the crown of the head to the navel anteriorly, and half way down the back, was raised into blisters of various sizes, which

---

* I do not mean to affirm or assert, that Dr. K. is the first who discovered and employed the oil of turpentine as a remedy in cases of burns, as it is mentioned by Dr. James, in his Medicinal Dictionary, so long since as the year 1743. After enumerating many remedies for burns, "he says, oil of turpentine also is to be had in readiness, with which the part is to be timely and frequently anointed."

Heister also mentions it in his Surgery, which was published in the year 1743.

which were filled with a deep yellow liquor, and which shews the severity of the scald. The parts which suffered the most injury were the face, breasts, and arms; but more particularly underneath the arms, from the axilla to the elbow, for there the scalding water insinuating into the cloaths, continued to be applied for a longer time to the skin. These parts, in a great measure, resembled burns more than scalds, as they assumed the appearance of large eschars, and in the course of the cure, extensive sloughs were thrown off.

The face in a few days put on the appearance, resembling very much the face of a person full of the worst kind of confluent small-pox; as it was covered with one continued dark brown coloured crustaceous mass, which formed a complete mask. She was nearly blind from the swelling and inflammation of the upper and lower palpebræ.

The accident happened about eleven o'clock in the forenoon. A surgeon was immediately sent for, who on his arrival ordered oil and water to be blended together, and then directed that linen cloths should be dipped into this liquid, and applied frequently to the parts affected. This application was continued eight hours, but without any apparent advantage.

At eight o'clock in the evening the patient was brought to the Infirmary, and after examining her, I found the extent of the injury from the scald to be what I have before stated; but with the addition of the following symptoms, which I did not then mention. Violent pain of the head, nausea at the stomach, and frequent efforts to vomit; the tongue was foul, continual shiverings similar to those of a person in the cold stage of an ague, and the pulse was quick. There was also great anxiety, and violent burning and smarting pains in the injured parts, which caused the patient to be so exceedingly restless, that it was difficult to keep her in bed. This was the state of the patient when I first saw her at the Infirmary.

I shall now proceed to detail the indications that were adopted at the onset, and in regular order, others, that were prescribed at different stages of the disease, until the cure was completed.

Externally. I first let out the contents of all the blisters.* I then dipped pieces of soft old linen into *cold* rectified

* Much has been said in regard to the propriety, or the impropriety, of opening the blisters caused by burns or scalds. Some authors say that they should tified oil of turpentine, wrung them out, and applied them over the scalded parts, and secured them by flannel rollers. The effect was agreeably astonishing, for she was no sooner dressed up, than she almost immediately fell into a comfortable sleep, and slept for nearly three hours. When she awoke she expressed herself in words truly thankful for such speedy relief, and said she was then much easier.

On account of the disordered state of the stomach, no medicines were administered except opiates, until the morning of the second day. She then began with a purging mixture, composed of a solution of the bitter purging salt in infusion of senna, then repeated as occasion required. And the patient now began to take every four hours, a saline draught, to which was added vin. antimon. gtt. xv. Tinct. opii. gtt. viii.

The first dressings were not removed until the morning of the third day; neither was it necessary to repeat the application

---

should be let out when first raised, others say not until the inflammation has subsided; but I should give the preference to the former opinion, as I apprehend the same effects take place to a certain degree, in vesications by burns, as does in those blisters which are produced by erysipelatous inflammation preceding a gangrenous tendency of the parts. Therefore the longer this ichorous liquor is suffered to remain, the more it injures the delicate and tender surface within. I have very often noticed that when such blisters have remained entire for several days, that on being opened with a lancet, or burst by accident, the parts within have always been more or less in a state of ulceration. But this effect seldom takes place if the blisters, in the first instance, are carefully punctured in the most dependant part, with the point of a narrow made lancet, or any similar instrument, leaving the bag or skin of the blister as a covering to guard the part from the external air, and afterwards covering the whole with such a dressing as may, according to circumstances, be thought most suitable. And it will not appear inconsistent to suppose that after caloric, either in a solid or a liquid form, has been applied to any part of the body, and where it has had the effect of raising blisters, that the serum thrown out by the exhalant vessels, must in consequence be rendered in a higher or lower degree more acrid, according to the degree and duration of heat which has been applied, producing such effects. Therefore I conceive the sooner this acrid serum is evacuated, the better it is for the patient, and the parts thus affected. Indeed, I need not extend this part of the subject any further than to illustrate this hypothesis by mentioning the effects produced by a common blister raised by cantharides. After a blister is fully formed by such a plaister, the patient commonly complains of a smarting and scalding pain, caused, as he says, by the water in the blister, and that the pain is always increased upon motion, such as turning in bed, or walking gently about, which diffuses the serum over the raw surface of the cutis vera, and produces pain, which certainly shews the acrid quality of the serum; but as soon as the contents of the blister is properly evacuated, and the part is dressed with a mild digestive ointment, the patient experiences immediate relief.

cation of the oil of turpentine a second time. At the first dressing, the parts affected assumed a better aspect than might a priori have been expected, considering the severity and extent of the injury. The parts were now dressed with the following ointment, spread upon soft old linen. ℞. Ung. resin. flav. unc. iii. ol. olivar. opt. ol. terebinth. rect. aa unc. 1. These, after being slowly melted together, should be stirred till quite cold. I have found these proportions of the different articles, to agree better with burnt and scalded parts, and less irritating to the patient, than equal parts of bazilicon and oil of turpentine, which I believe is the liniment used and recommended by Dr. Kentish.

On the fourth day there was evident signs of suppuration* commencing; therefore, instead of applying the warm dressing to the parts least injured, the following cerate was made use of, which agreed very well with the sores, and healed them up rapidly. ℞. Cerat. lap. calam. unc. 1. plumb. ust. rubr. dr. 1. misce. The warm dressing was still continued to the parts that were foul and sloughy, until the sloughs came away, and then the ulcers were dressed in a common way.†

In

---

* In many places there was merely a separation of the cuticle, yet from these parts, there was a copious discharge of well concocted pus, many days: had Hildanus seen this case, he would have been no less surprised at such an effect, than he was with the case of a maid who had scalded her whole leg up to the thigh with water, and from whence half a pound of matter was discharged at each dressing, twice a day. See Swieten's Commentaries upon Boerhaave's Aphorisms, vol. 4, page 193.

† Dr. Kentish says, "as for the dead parts, the application which is immediately applied to them is of very little consequence, for the throwing off of these eschars depends upon a process of the system, which the immediate application to the dead part will in no way either retard or facilitate." I am of a different opinion, and need only mention in support of it, and by way of example, an eschar that is produced by the application of a common caustic, the separation of which from the living parts, I can affirm with truth may be greatly facilitated by the application of warm and emollient dressings. And Dr. K. in his second Essay upon burns, in some degree contradicts his former assertion. He says, "in cases of eschars coming away, or while they are detaching themselves, I fill up the hollow made by their loss when separated, and fill up their furrow at their edges when loosening, with powdered chalk, which is covered with a plaister of cerate; and if the process be tedious, a poultice of bread and milk is applied over the plaister?

In cases of sloughs and eschars, the animal œconomy always furnishes what is termed the suppurative stage, which is the process instituted by nature for the purpose of separating the unsound or dead parts from the sound or living parts, therefore I do not see the utility of absorbing the secretion

In consequence of the difficulty to retain any dressings upon the face, it was anointed three times a day with a little of the following liniment, applied with the end of a soft feather. ℞. Ol. oliv. opt. aq. calcis. aa unc. 1. Aq. litharg. acet. dr. ii. misce.

The draughts before mentioned were continued until the 7th of November, with very great advantage; but as the heat of the body and the pains were now subdued, excepting at the times of dressing, they were left off; and the patient began to take three table spoonsful of the decoction of bark, every six hours. The bark, with laxatives occasionally, milk, and nourishing broths, were persisted in from the 7th of November until the 19th of December, when the girl was discharged cured.

### *Further Remarks upon the Case.*

It was observed before, that the patient, previous to her being scalded, had been subject to frequent attacks of epileptic fits. She called upon me at the Infirmary eight weeks after her dismissal, to shew me those parts upon her arms that had suffered the most from the scald, with an idea that they were going to gather, and be bad again; but upon examining them, I found that large seams, or cicatrices, which are common after such accidents, had formed, and were considerably elevated above the surface of the sound skin adjacent to these parts. It

---

cretion of matter with chalk, as it forms in the furrow surrounding the eschar, because it must in some degree retard its separation; and the application of a plaister and a poultice over the chalk, appear to be remedies diametrically opposite to each other, as poultices are generally intended to act as a stimulus by drawing a gentle heat to the part, and consequently must assist in the process of suppuration.

Dr. Kentish in his second pamphlet also mentions that he uses chalk for the purpose of repressing the growth of fungus, and of absorbing the redundant secretion; and presently after it appears as if he used chalk, whether the secretion be redundant or not; for he says, "as soon as secretion takes place, I begin the use of powdered chalk heated to the temperature of the human body, which is plentifully applied to the whole secreting surface, and afterwards covered with a plaister of cerate. If it be a process of the system to produce matter beneath and round the sides of sloughs and eschars, in order to facilitate their separation from the sound parts; why should this discharge, in its very commencement, be absorbed where it is so essentially necessary, unless it be really over abundant in quantity?

I have tried powdered chalk in cases of chronic ulcers left by burns and scalds, where there was a copious discharge, and a good share of fungus. The secretion was diminished by it, but the fungus rather increased during its application, I therefore discontinued the use of it, and have applied other more active escharotics, such as alumen ustum, zincum vitriolatum,—calcinatum, cuprum vitriolatum, and the argentum nitratum.

It is recorded that a violent head-ache in one person, and a pain in the limbs in another person, were removed by the parts affected being accidently burnt, and that only in the first degree of burns.* And it deserves to be mentioned, that this girl has had no return of the epileptic fits since the time of her being scalded, which is now nearly four months ago. However, upon further enquiry into her then present state of health, I learned that she had not yet had the catamenia; but that she had been regularly attacked, about the end of every third week, with violent pain of the head, and vomitings of bile, which always continues, more or less, for two or three days; then these symptoms go off, and she remains well until the next attack, which is an interval of about three weeks. I conceive these complaints to be symptomatic of chlorosis, which no doubt will continue to trouble her till a change † is produced in the uterine vessels, causing a regular discharge of the menses.

In regard to the scald in this case having removed the epilepsy, it accords with many other instances of this nature, which are recorded by different authors. One case, somewhat similar to this, is mentioned in Dr. Kentish's first Pamphlet, see page 67.

Homberg thinks that burning with moxa, with cauteries, &c. cure by quickening the motion of the humours, and thinning them, and by destroying the ends of the vessels by which the humours flow less that way. Hence appears the great utility of caustics, setons, blisters, &c. in the treatment of various diseases. I am, &c.

*Oct.* 2, 1807. ROBERT EARNEST,

House Surgeon to the Sheffield General Infirmary.

---

* See Motherby's Medical Dictionary, Art. AMBUSTA.

† I am happy that it is in my power to say, that this change has taken place. I met with the young woman's aunt by accident, a few days ago, who informed me that three months since, her niece had been attacked several times by epistaxis; which seemingly was the harbinger of the catamenia, which took place almost immediately after blood ceased to flow from the nose, and the natural discharge has been regularly evacuated, for the last three months; and the girl is now in perfect health.

It will not be improper again to remark that upwards of ten months have now elapsed, and there has been no recurrence of the epileptic fits.

